# Neurodegeneration Within the Amygdala Is Differentially Induced by Opioid and HIV-1 Tat Exposure

**DOI:** 10.3389/fnins.2022.804774

**Published:** 2022-05-04

**Authors:** Sara R. Nass, Michael Ohene-Nyako, Yun K. Hahn, Pamela E. Knapp, Kurt F. Hauser

**Affiliations:** ^1^Department of Pharmacology and Toxicology, Virginia Commonwealth University, Richmond, VA, United States; ^2^Department of Anatomy and Neurobiology, Virginia Commonwealth University, Richmond, VA, United States; ^3^Institute for Drug and Alcohol Studies, Virginia Commonwealth University, Richmond, VA, United States

**Keywords:** amyloid-β, tauopathy, tau phosphorylation isotypes, pSer396, pThr205, neurofibrillary tangles, HIV-associated neurocognitive disorders (HAND), chronic opioids

## Abstract

Opioid use disorder (OUD) is a critical problem that contributes to the spread of HIV and may intrinsically worsen neuroHIV. Despite the advent of combined antiretroviral therapies (cART), about half of persons infected with HIV (PWH) experience cognitive and emotional deficits that can be exacerbated by opioid abuse. HIV-1 Tat is expressed in the central nervous system (CNS) of PWH on cART and is thought to contribute to neuroHIV. The amygdala regulates emotion and memories associated with fear and stress and is important in addiction behavior. Notwithstanding its importance in emotional saliency, the effects of HIV and opioids in the amygdala are underexplored. To assess Tat- and morphine-induced neuropathology within the amygdala, male Tat transgenic mice were exposed to Tat for 8 weeks and administered saline and/or escalating doses of morphine twice daily (s.c.) during the last 2 weeks of Tat exposure. Eight weeks of Tat exposure decreased the acoustic startle response and the dendritic spine density in the basolateral amygdala, but not the central nucleus of the amygdala. In contrast, repeated exposure to morphine alone, but not Tat, increased the acoustic startle response and whole amygdalar levels of amyloid-β (Aβ) monomers and oligomers and tau phosphorylation at Ser396, but not neurofilament light chain levels. Co-exposure to Tat and morphine decreased habituation and prepulse inhibition to the acoustic startle response and potentiated the morphine-induced increase in Aβ monomers. Together, our findings indicate that sustained Tat and morphine exposure differentially promote synaptodendritic degeneration within the amygdala and alter sensorimotor processing.

## Introduction

The opioid epidemic is a public health crisis that is increasing the rise of viral infections, such as HIV-1 attributed to injected drug use (Hodder et al., 2021). Further, persons infected with HIV (PWH) are more likely to be prescribed opioids for pain management than HIV negative individuals (Silverberg et al., 2012; Edelman et al., 2013). Although combination antiretroviral therapy (cART) has increased the lifespan and quality of life of PWH, many still develop HIV-associated neurocognitive disorders (HAND) that can be exacerbated by opioid use (Bing et al., 2001; Byrd et al., 2011, 2012; Meyer et al., 2013; Paydary et al., 2016). Further, the regulatory HIV-1 protein, trans-activator of transcription (Tat) is present in the cerebrospinal fluid (CSF) of PWH despite cART (Johnson et al., 2013; Henderson et al., 2019). Many of the cognitive and behavioral deficits and region-specific neuronal dysfunction, injury, and inflammation seen in PWH can be recapitulated in Tat-transgenic (tg) mice, suggesting a fundamental role of Tat in HAND neuropathogenesis (Fitting et al., 2013; Hahn et al., 2015; Kesby et al., 2016; Marks et al., 2016; McLaughlin et al., 2017; Gonek et al., 2018; Nookala et al., 2018; Cirino et al., 2020; Nass et al., 2020; Paris et al., 2021).

Synaptodendritic injury correlates with the development of neurocognitive deficits seen in HAND patients (Ellis et al., 2007; Irollo et al., 2021), similar to Alzheimer’s disease (AD) (Dorostkar et al., 2015). Moreover, neurotoxic amyloid-β (Aβ) aggregates, hyperphosphorylated tau, and neurofilament light chain (NFL), characteristic biomarkers of AD, are found in the central nervous system (CNS) of PWH in a region-specific manner (Andersson et al., 1999; Gelman and Schuenke, 2004; Brew et al., 2005; Green et al., 2005; Ramage et al., 2005; Anthony et al., 2006; Achim et al., 2009; Gisslen et al., 2009; Peterson et al., 2014; McGuire et al., 2015; Krut et al., 2017). Interestingly, Aβ levels in the CSF of PWH with HAND, although variable (Gisslen et al., 2009; Peterson et al., 2014), tend to be reduced compared to neuroasymptomatic PWH and HIV negative controls (Brew et al., 2005; Clifford et al., 2009; Dorostkar et al., 2015), which could be due to an accumulation of Aβ deposits in the brain (Fagan et al., 2007, 2009). Similarly, individuals with opioid use disorder (OUD) exhibited increased frontal cortical, hippocampal, and brain stem Aβ plaques and hyperphosphorylated tau compared to controls (Ramage et al., 2005; Anthony et al., 2010; Kovacs et al., 2015). Nevertheless, the comorbid effects of HIV and opioids, and regional differences in the accumulation of these neurodegenerative markers are underexplored.

The amygdala is important for emotional and reward processing, and associated memory formation, as well as sensorimotor gating (Decker et al., 1995; Wan and Swerdlow, 1997; LeDoux, 2007; Nikolenko et al., 2020; Cano et al., 2021). Its role in emotional saliency makes the amygdala a key mediator of addiction behavior—particularly those behaviors associated with drug reinforcement and the aversive effects of withdrawal (Koob and Le Moal, 2001; Koob and Volkow, 2010). Functional connectivity between the amygdala and prefrontal cortex (PFC), insula, and striatum are decreased (Upadhyay et al., 2010; Zhang et al., 2017), while amygdalar and hippocampal associations are increased (Zhang et al., 2017) in individuals with OUD, suggesting a loss of inhibitory control and an increased consolidation of maladaptive memory and learning, respectively. Individuals with OUD also tend to have smaller amygdalar volumes compared to controls (Upadhyay et al., 2010; Younger et al., 2011).

PWH can also exhibit reduced amygdalar volume (Ances et al., 2012; Kallianpur et al., 2013), that is worsened by experiences of increased social adversity (Thames et al., 2018) and decreased resting-state connectivity between the amygdala and PFC compared to healthy controls (McIntosh et al., 2018; Philippi et al., 2020). Further, in PWH on cART, there is a negative correlation between viral load and amygdalar volume (Nir et al., 2021). By contrast, others find increased amygdalar volume in PWH compared to healthy controls, even when controlling for drug use and socioeconomic factors (Clark et al., 2012, 2015). Given these disparate outcomes in PWH, we decided to examine amygdala-associated behavioral deficits and tissue-level neuropathology in a more controllable, preclinical model.

We previously showed that HIV-Tat and morphine induce synaptodendritic injury (Fitting et al., 2010, 2013; Hahn et al., 2015; Marks et al., 2016; Nass et al., 2020) and increase levels of pathologically phosphorylated tau (p-tau) (Ohene-Nyako et al., 2021) in a region-dependent manner. Similar pathological effects have not been explored in the amygdala. We hypothesized that HIV-1 Tat and morphine would decrease prepulse inhibition (PPI) of the acoustic startle response and amygdalar spine density while increasing biomarkers of neurodegeneration. Eight weeks of Tat exposure decreased, whereas morphine increased the acoustic startle response. Further, Tat and morphine combined to decrease habituation and PPI of the acoustic startle response, mimicking the impairments in individuals with neurodegenerative and cognitive disorders, including PWH (Minassian et al., 2013; Walter et al., 2021). Tat also decreased the dendritic spine density in the basolateral amygdala (BLA), but not in the central nucleus of the amygdala (CeA) in Tat-tg mice. Alternatively, repeated administration of morphine by itself increased whole amygdalar levels of Aβ monomers and oligomers, and the tau phospho-isotype pSer396.

## Materials and Methods

### Subjects and HIV-Tat Induction

**FIGURE 1:**
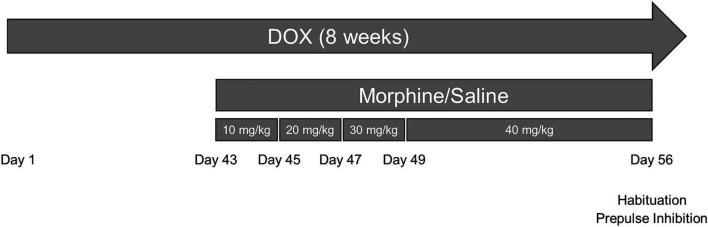
Experimental design depicted on a timeline. Tat transgenic mice received doxycycline (DOX)-containing chow for 8 weeks (the entire experimental length) and were repeatedly injected with morphine (10–40 mg/kg, increasing by 10 mg/kg/2 day, s.c., b.i.d.) or saline for the last 2 weeks of DOX administration. On day 56 (day 14 of repeated morphine injections), mice were tested for the acoustic startle response, habituation to the startle tone, and prepulse inhibition (PPI) of the response. On day 57, the day following behavioral testing, mice were euthanized, and tissues were randomly assigned to immunoblotting or Golgi assays.

Doxycycline (DOX)-inducible GFAP-driven *tet*-on HIV-1_*IIIB*_ Tat_1–86_ transgenic mice (Bruce-Keller et al., 2008; Nass et al., 2020, 2021) express Tat mRNA and/or protein within 48 h of DOX administration throughout the CNS (e.g., cortex, hippocampus, striatum, and spinal cord) (Bruce-Keller et al., 2008; Fitting et al., 2010, 2012; Carey et al., 2012; Dickens et al., 2017). Adult (3–5-month-old) male Tat-tg mice (*n* = 64) were generated in the vivarium of Virginia Commonwealth University and were housed 3–4 per cage in a temperature- and humidity-controlled, AAALAC-accredited facility, on a 12:12 light:dark cycle. Mice were fed a standard chow supplemented with doxycycline (DOX, 6 g/kg, Harlan Laboratories, Madison, WI) for 8 weeks to induce CNS expression of HIV-1 Tat in Tat(+) mice and control for off-target effects in Tat(–) mice with *ad libitum* access to food and water (Figure 1). The Virginia Commonwealth University Animal Care and Use Committee approved the use of mice in these studies and all experiments were conducted in accordance with the National Institutes of Health (NIH Publication No. 85–23) ethical guidelines.

### Drug Treatment

Morphine sulfate (National Institute on Drug Abuse, Bethesda, MD) was dissolved in sterile saline and mice were administered escalating doses of morphine (10–40 mg/kg, increasing by 10 mg/kg/2 day, s.c.) twice daily (b.i.d.) or saline vehicle for the last 2 weeks of DOX administration as previously described (Nass et al., 2021; Ohene-Nyako et al., 2021). Therefore, mice received 10 mg/kg on days 1 and 2, 20 mg/kg on days 3 and 4, 30 mg/kg on days 5 and 6, and 40 mg/kg on days 7–14 or saline starting at 6 weeks following Tat induction (Figure 1). All solutions were administered at room temperature at a volume of 10 μl/g body weight.

### Prepulse Inhibition of the Acoustic Startle Response

The amygdala is an important component of prepulse inhibition (PPI) of the acoustic startle response (Cano et al., 2021). PPI is a measure of sensorimotor gating that is decreased in many neurocognitive disorders and corresponding animal models (Swerdlow et al., 2001, 2016). As previously described (Nass et al., 2020), for 2 days before testing, mice were habituated to the startle response chamber and restrainer (Kinder Scientific Startle Monitor, Poway, CA) with a 65 decibel (dB) background noise. On the testing day, mice were habituated to the testing room for at least 1 h. During the acoustic startle response trial blocks, mice were subjected to a startle tone of 120 dB for 64 trials with randomized ITIs averaging 15 s. During the PPI blocks, lower-decibel (dB) non-startling prepulse tones (4, 8, or 16 dB above background) were presented 100 ms before the startle pulse. The acoustic startle response, habituation, and PPI were measured as the force exerted in Newtons (N) when jumping/responding to the startle tone. To control for any differences in body weight that may influence jumping force, body weight was assessed as a covariate.

### Dendritic Spine Density Assessment

Golgi impregnations were performed according to manufacturer’s directions (FD NeuroTechnologies, Columbia, MD), as previously described (Nass et al., 2020). After 8 weeks of DOX exposure, 2 weeks of ramped morphine, and behavior assessment, Tat(+) and Tat(–) mice were humanely euthanized by rapid cervical dislocation. Whole forebrains were harvested, and Golgi-impregnated using the proprietary solutions supplied. Sections were then cut (185 μm-thick) in the coronal plane using a Leica VT1000s vibratome (Leica Biosystems, Wetzlar, Germany), mounted on gelatin-coated glass slides, stained, dehydrated in graded ethanol solutions, cleared in xylene, and coverslipped with Permount. The Mouse Brain in Stereotaxic Coordinates (Franklin and Paxinos, 2008) atlas was used to locate fully impregnated neurons within the BLA and CeA of the amygdala. The number of spines along the tertiary dendrites of glutamatergic pyramidal neurons in the BLA and GABAergic medium spiny-type neurons in the CeA were counted using a Zeiss Axio Examiner D1 upright microscope (Zeiss, Oberkochen, Germany) and reported as the number of spines/10 μm dendrite in accordance with previous methods (Fitting et al., 2013; Hahn et al., 2015; Nass et al., 2020). The identity of the neuronal cell types in each nucleus was confirmed by morphological criteria (McDonald, 1982; Tosevski et al., 2002).

### Immunoblotting

Biomarkers of neurodegeneration were assessed in the amygdala of Tat(+) and Tat(–) mice after administration of DOX for 8 weeks and escalating doses of morphine in some groups during the last 2 weeks of DOX exposure, and behavioral assays, as previously described (Nass et al., 2020; Ohene-Nyako et al., 2021) with minor modifications. Mice were humanely euthanized by rapid cervical dislocation. The amygdala was dissected, snap-frozen in liquid nitrogen, and stored at –80°C until use. Tissues were homogenized in Pierce IP lysis buffer (Thermo Fisher Scientific, Waltham, MA) containing Halt protease and phosphatase inhibitor cocktail (Thermo Fisher Scientific) and protein levels were quantified using the bicinchoninic acid (BCA) assay (Pierce Biotechnology, Rockford, IL). Tissue lysates (30 μg) were loaded into 4–20% Criterion TGX Stain-Free gels (Bio-Rad, Hercules, CA), separated by electrophoresis under denaturing and reducing conditions, and transferred to Immuno-Blot PVDF membranes (Bio-Rad). Membranes were blocked for 1 h with Intercept (TBS) Blocking Buffer (#927-60001 Li-Cor, Lincoln, NE) and then incubated overnight with primary antibodies to Aβ (#ab201060, Abcam, Boston, MA; 1:1,000), tau (#46687, Cell Signaling Technology, Danvers, MA; 1:2,000), pSer396 (#9632 Cell Signaling Technology; 1:1,000), pThr205 (#49561 Cell Signaling Technology; 1:1,000), and NFL (#ab7255, Abcam; 1:1,000), followed by species-appropriate fluorescent secondary antibodies. Immunoblotted proteins were visualized on a ChemiDoc MD imaging system and analyzed using Image Lab 5.2.1 (Bio-Rad). pSer396 and pThr205 levels were ratioed to total tau levels and all protein levels were normalized to GAPDH (Abcam; 1:2,500). Data are presented as the percentage change from control values (i.e., Tat(–) mice that received saline).

### Immunohistochemistry

The cell-type specific distributions of MOR in the BLA and CeA were visualized using immunohistochemistry. Tat(–)/saline (control) mice were transcardially perfused with 4% paraformaldehyde (PFA, pH 7.4; Sigma-Aldrich Co., St. Louis, MO) in phosphate-buffered saline (PBS) and whole forebrains were removed. Fresh PFA was used to post-fix brains overnight at 4°C. Brains were then rinsed in PBS and submerged in 10 and 20% sucrose sequentially for at least 24 h each before being embedded in Tissue-Tek O.C.T. compound (Sakura Finetek, Torrance, CA) on dry ice and stored at –80°C. Sections containing the BLA and CeA were cut coronally at a thickness of 16 μm on a Leica CM1850 cryostat and mounted on SuperFrost Plus Gold slides (Thermo Fisher Scientific). Slides were warmed to room temperature and dried for 15 min. Sections were rehydrated in PBS, incubated in permeability solution (0.2% Triton X-100 in PBS) for 30 min, and incubated in blocking solution (1% normal donkey serum + 1% bovine serum albumin in PBS) for 2 h. Primary antibodies against GAD67 (chicken polyclonal ab75712, Abcam; 1:200), MOR (rabbit monoclonal cat. no. AOR-011, Alomone Labs, Jerusalem, Israel; 1:200), and NeuN (Mouse monoclonal cat. no. MAB377, Millipore, Burlington, MA; 1:200) were diluted in blocking buffer, and sections were incubated in the primary antibody solution at 4°C for 48 h. Sections were washed in PBS and incubated for 1 h at room temperature with appropriate, fluorescently labeled secondary antibodies conjugated to Alexa 488 (donkey anti-chicken, Invitrogen; 1:200), Alexa 594 (donkey anti-rabbit, Invitrogen; 1:200), or Alexa 647 (donkey anti-mouse, Invitrogen; 1:200). Hoechst 33342 (Invitrogen; 1:20,000) was used to identify nuclei. Sections were again washed in PBS and mounted with coverslips in ProLong Gold Antifade reagent (Invitrogen). The BLA and CeA from tissue sections (*n* = 3) were imaged using a Zeiss LSM 700 confocal microscope at 63 × magnification (Zeiss, Oberkochen, Germany).

### Statistical Analysis

Behavioral measures were assessed by 2-way analysis of covariance (ANCOVA) (acoustic startle response) or repeated measures 2-way ANCOVA (habituation and PPI) with genotype and morphine exposure as between-subjects factors, body weight as a covariate, and the startle response trial block or intensity of the prepulse tone in dB as the within-subject factor, respectively, using SPSS Statistics 27.0 (IBM, Armonk, New York). Spine density and immunoblotting data were analyzed by 2-way analysis of variance (ANOVA) (Tat genotype × morphine treatment) using Prism version 9.2 (GraphPad Software, San Diego, CA). *A priori* planned comparisons between groups were performed with Bonferroni corrections for multiple comparisons to assess Tat and morphine interactions.

## Results

### HIV-1 Tat and Morphine Differentially Alter the Acoustic Startle Response, Habituation, and Prepulse Inhibition

**FIGURE 2:**
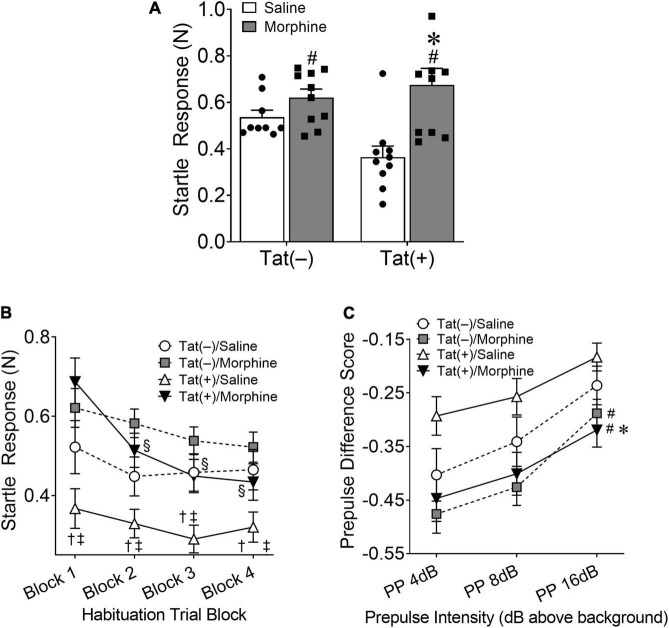
HIV-1 Tat decreases, whereas morphine increases the acoustic startle response. Tat(+) and Tat(–) mice were given DOX chow for 8 weeks and received ramping morphine (or saline) during the last 2 weeks of exposure. In the acoustic startle response test, repeated morphine increased the startle response **(A)**. In contrast, Tat decreased the startle response in saline-treated mice **(A)**. During habituation testing Tat(+)/saline (trial blocks 3 and 4) and morphine (across all trial blocks) mice exhibited significantly higher startle responses compared to Tat(–)/saline mice **(B)**. However, only Tat(+)/morphine mice exhibited habituation to the startle response **(B)**. Further, in combination, Tat and morphine decreased the prepulse inhibition (PPI) to the acoustic startle response **(C)**. Data are presented as the mean ± the SEM; *n* = 9–10 mice per group; *p < 0.05, pairwise comparison difference from Tat(+)/saline; ^†^*p* < 0.05, pairwise comparison difference from Tat(–)/morphine; ^‡^*p* < 0.05, pairwise comparison difference from Tat(+)/morphine; ^§^*p* < 0.05, Tat(+)/morphine significantly decreased from block 1; ^#^*p* < 0.05, main effect of morphine. N, newton; dB, decibel.

First, we tested the acoustic startle response, followed by habituation and PPI of the response after 8 weeks exposure to Tat with escalated morphine during the last 2 weeks. A significant main effect indicated that morphine increased [*F*_(1, 34)_ = 11.92, *p* < 0.01; Figure 2A] the initial startle response to a loud tone. There was also an interaction between Tat and morphine in the acoustic startle response [*F*_(1, 34)_ = 4.77, *p* < 0.05; Figure 2A]. *Post hoc* comparisons showed that Tat(+)/saline mice exhibited a decreased startle response compared to Tat(–) and Tat(+) morphine treated mice (*p* < 0.01). When assessing habituation to the startle tone, there was a significant interaction between Tat expression, morphine treatment, and habituation trial block [*F*_(3, 102)_ = 3.327, *p* < 0.05; Figure 2B]. *Post hoc* comparisons revealed that Tat(–)/saline mice exhibited significantly lower startle responses compared to Tat(+)/saline mice in blocks 3 and 4 (*p* < 0.05) and morphine-treated Tat(–) and Tat(+) mice across all blocks (*p* < 0.05). However, only Tat(+)/morphine mice exhibited habituation across the 4 blocks of the startle response (*p* < 0.05). In the PPI assessment, there was a significant interaction between Tat expression and dB level above background [*F*_(2, 68)_ = 3.752, *p* < 0.05; Figure 2C] and a main effect of morphine treatment [*F*_(1, 34)_ = 8.168, *p* < 0.01; Figure 2C] on PPI responses. Pairwise comparisons indicated that combined Tat and morphine exposure decreased the PPI of the acoustic startle response compared to Tat(+)/saline-administered mice at all dB levels tested, suggesting differential effects of sensorimotor gating.

### HIV-1 Tat, but Not Morphine Decreases Dendritic Spine Density in Distinct Amygdalar Nuclei

**FIGURE 3:**
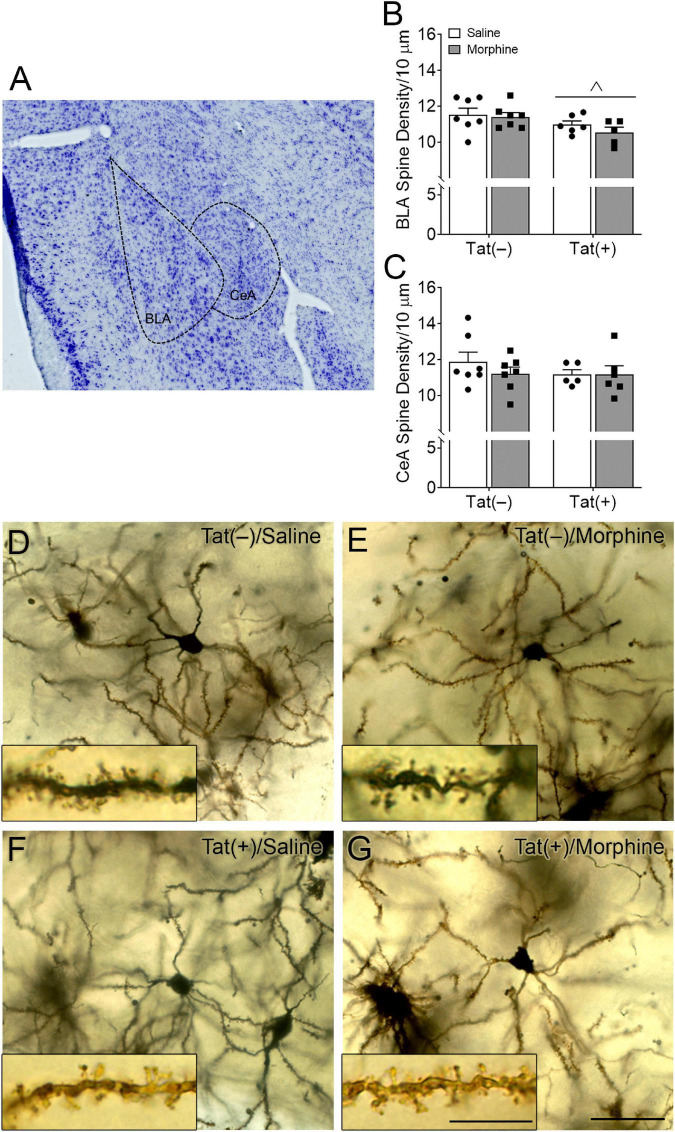
HIV-1 Tat decreased dendritic spine density in the basolateral, but not central nuclei of the amygdala. Representative Nissl-stained image showing the basolateral (BLA) and central (CeA) nuclei of the amygdala **(A)**. Tat exposure for 8 weeks, regardless of repeated morphine exposure during the final 2 weeks of the experiment, decreased the dendritic spine density of pyramidal neurons in the BLA **(B)**, but not the density of dendritic spine in medium spiny-type neurons within the CeA **(C)**. Representative images of Golgi-impregnated BLA pyramidal neurons **(D–G)** (scale bar = 50 μm). Appearance of pyramidal neurons and tertiary (3rd order; scale bar = 10 μm) dendrites in the BLA following saline **(D)** or morphine **(E)** administration in Tat(–) mice, and saline **(F)** and morphine **(G)** administration in Tat(+) mice. Data are presented as the mean ± the SEM; multiple neurons (≥6) were sampled in each *n* = 5–7 mice per group, ^∧^*p* < 0.05, main effect of Tat.

Within the subdivisions of the amygdala, the BLA is considered the main input nuclei, whereas the CeA is the output region (LeDoux, 2007; Nikolenko et al., 2020). To assess synaptic culling or injury within the amygdala, we measured the spine density in tertiary dendrites of Tat-tg and control mice ± morphine exposure. Regardless of morphine treatment, Tat decreased the spine density of glutamatergic pyramidal neurons in the BLA [*F*_(1, 21)_ = 6.13, *p* < 0.05; Figures 3A,B,D–G]. There was no effect of Tat or morphine on the density of spines in tertiary dendrites of GABAergic medium spiny-type neurons in the CeA (*p* = 0.47; Figures 3A,C).

### HIV-1 Tat and Morphine Differentially Alter Amygdalar Levels of Neurodegenerative Biomarkers

**FIGURE 4:**
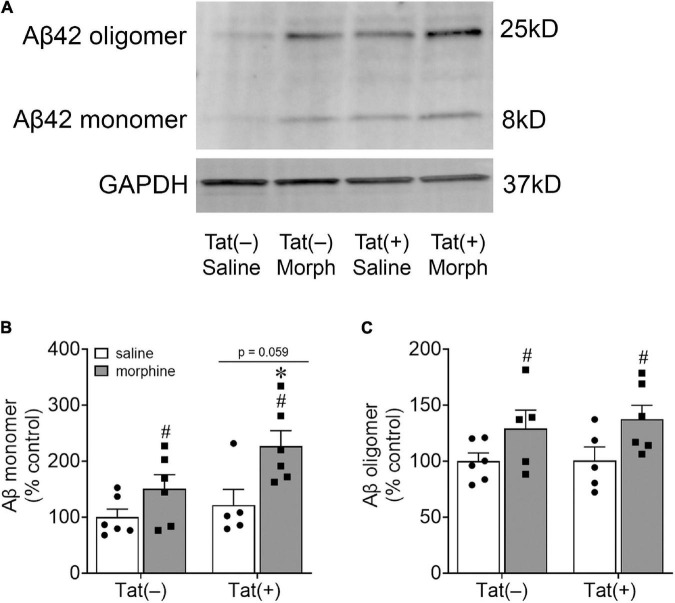
Morphine increased amygdalar levels of amyloid-β. Representative images of amyloid-β (Aβ) monomer and oligomer immunoblots within the amygdala of Tat-tg mice that received DOX for 8 weeks and ramped doses of morphine during the final 2 weeks of the experiment **(A)**. A main effect indicated that morphine exposure increased Aβ monomer levels in the amygdala, while Tat tended to increase Aβ monomer levels (*p* = 0.059) **(B)**. Pairwise comparisons suggest that this effect was partially driven by the Tat(+) mice administered morphine **(B)**. Morphine, but not Tat, exposure also increased amygdalar Aβ oligomer levels **(C)**. *p < 0.05, pairwise comparison difference from Tat(+)/saline; ^#^*p* < 0.05, main effect of morphine.

**FIGURE 5:**
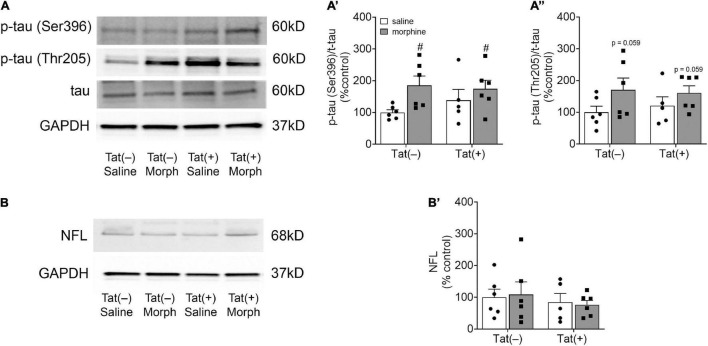
Morphine increased amygdalar levels of the tau phospho-isotype pSer396. Representative images of amygdalar pTau (Ser396), pTau (Thr205), and total tau **(A)**, and neurofilament light chain (NFL) immunoblots **(B)** of Tat(–) and Tat(+) mice administered DOX for 8 weeks and ramping morphine (or saline) during the last 2 weeks of exposure. In both Tat(–) and Tat(+) mice given ramping morphine for 2 weeks the ratio of p-tau (Ser396) to total tau was significantly increased **(A’)**, while the ratio of p-tau (Thr205) to total tau tended to be elevated (albeit not significantly) **(A”)**. Neither Tat nor morphine altered neurofilament light chain levels in the amygdala **(B’)**. Data are presented as the mean ± the SEM; *n* = 6–7 mice per group, ^#^*p* < 0.05 main effect of morphine. Morph, morphine; NFL, neurofilament light chain.

We next assessed amygdalar levels of the neurodegenerative biomarkers Aβ, phospho-isotypes of tau pSer396 and pThr205, and NFL, all of which are associated with cognitive deficits and dementia (de Wolf et al., 2020). Escalating morphine exposure for 2 weeks increased levels of Aβ oligomers (pathogenic) [*F*_(1, 18)_ = 7.34, *p* < 0.05; Figures 4A,C] and monomers (pre-pathogenic) [*F*_(1, 19)_ = 10.36, *p* < 0.01; Figures 4A,B] in the whole amygdala. A main effect showed that Tat also tended to increase amygdalar monomeric Aβ [*F*_(1, 19)_ = 4.03, *p* = 0.059; Figures 4A,B], and planned comparisons indicated that morphine increases monomeric Aβ in Tat(+) (*p* < 0.05), but not Tat(–) mice (*p* = 0.29). Similarly, repeated morphine increased the amygdalar ratio of pSer396 to total tau [*F*_(1, 19)_ = 5.34, *p* < 0.05; Figures 5A,A’], whereas the ratio of pThr205 to total tau tended to be increased by morphine [*F*_(1, 19)_ = 4.02, *p* = 0.059; Figures 5A,A”]. However, neither Tat nor morphine altered levels of NFL in the amygdala (*p* = 0.77; Figures 5B,B’).

### Qualitative Imaging of MOR Expression in the Basolateral Amygdala and Central Nucleus of the Amygdala Amygdalar Nuclei

**FIGURE 6:**
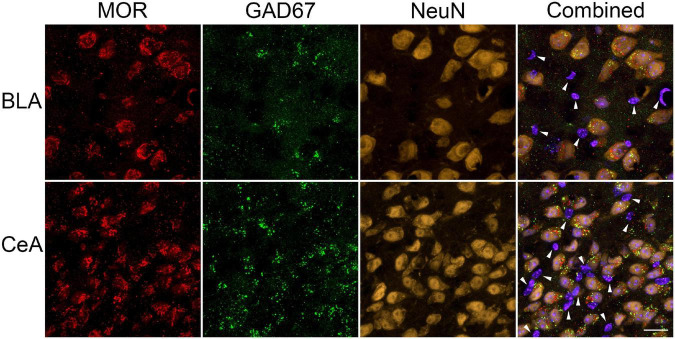
Cellular localization of μ-opioid receptor (MOR) expression in the amygdala. Representative images of GABAergic marker GAD67 (green), MOR (red), neuronal marker NeuN (yellow-orange), and Hoechst (violet-blue) positive cells were taken using a Zeiss LSM 700 microscope at 63 × magnification (Zeiss, Oberkochen, Germany). Most MOR immunoreactive-cells in the basolateral amygdala (BLA) and central nucleus of the amygdala (CeA) were positive for GAD67 and NeuN, suggesting MOR is predominately located on GABAergic neurons within these amygdalar nuclei. However, (as indicated by the white arrows) some MOR immunoreactive-cells did not colocalized with GAD67 nor NeuN. Scale bar = 20 μm.

Finally, we examined the expression of MOR on neurons within the BLA and CeA of the amygdala by colocalization of MOR with the neuronal marker NeuN and the GABAergic interneuron marker GAD67. Most neurons were positive for MOR and GAD67 in both amygdalar nuclei, suggesting MOR is present mainly on inhibitory interneurons vs. pyramidal neurons in the BLA (Figure 6). Although in the CeA, MOR could also be expressed on GABAergic medium spiny-type neurons (Figure 6). However, some MOR^+^ cells did not co-express NeuN suggesting MOR is also expressed on other cell types, such as astroglia.

## Discussion

**FIGURE 7:**
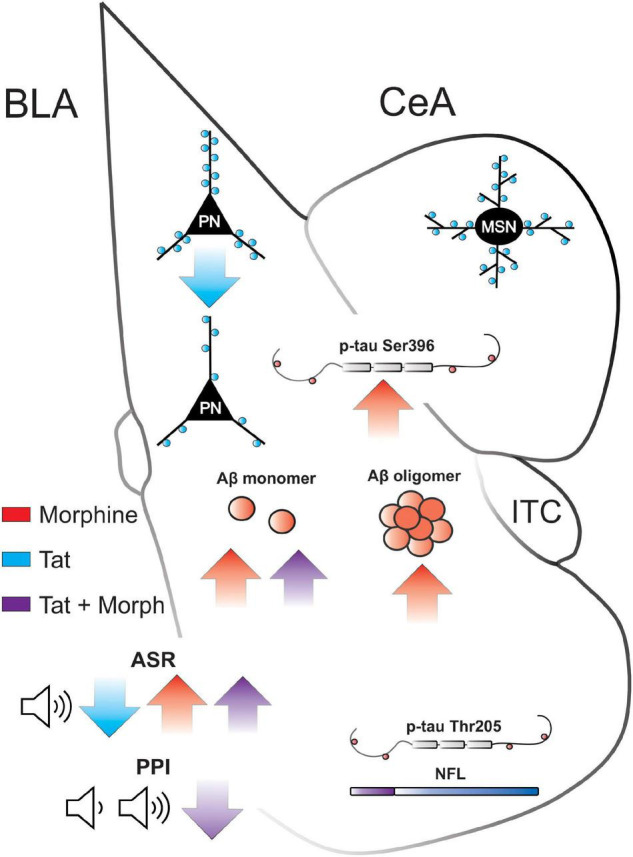
Summary of Tat and morphine-induced amygdalar deficits. BLA, basolateral amygdala; CeA, central nucleus of the amygdala; ITC, intercalated cells; PN, pyramidal neuron; MSN, medium spiny neuron; Aβ, amyloid-β; p-tau, phosphorylated tau; NFL neurofilament light; ASR, acoustic startle response; PPI, prepulse inhibition. ↑ or ↓ indicate significant increase or decrease, respectively, by Tat (red), morphine (blue), or the interaction of Tat and morphine (purple).

The amygdala is involved in emotional, reward, and memory processing—deficits are seen in PWH and OUD (Koob, 2021). The startle reflex and PPI are modulated by amygdalar function, particularly the BLA, and are altered in neuropsychiatric and neurodegenerative disorders, as well as in animal models (Angrilli et al., 1996; Wan and Swerdlow, 1997; Alsene et al., 2011; Forcelli et al., 2012; Diano et al., 2016). PWH exhibit decreased PPI of the acoustic startle response (Walter et al., 2021), indicating a decrease in sensorimotor processing, albeit in another study only PWH who also exhibited HAND showed a significant reduction in PPI (Minassian et al., 2013). The Tat-tg mouse line used in the current study expresses a single copy of the *tat* gene that induces a slower onset, chronic pathology resembling that exhibited by persons infected with HIV (PWH) (Dickens et al., 2017). However, in the present study, HIV-1 Tat decreased the acoustic startle response in saline treated mice, whereas habituation and PPI to the response were only decreased in Tat(+) mice administered morphine (summarized in Figure 7). Interestingly, we previously found that 8 weeks of Tat exposure did not alter the acoustic startle response but did decrease PPI at all prepulse tones (Nass et al., 2020). Further, in an alternative Tat-tg mouse line expressing 3–7 copies of the *tat* gene (Kim et al., 2003), Tat increased the acoustic startle response while decreasing PPI at 8 or 16 dB above baseline (Paris et al., 2015; Walter et al., 2021). Discrepancies in PPI results in the current study compared to previous studies with Tat, could be due to the accelerated neuropathogenesis in the model with more copies of the *tat* gene (Kim et al., 2003), which results in a more fulminating pathological progression. These mice display an accelerated pathology that is otherwise similar to our Tat-tg line but are less amenable to chronic studies as the pathology can be quite severe. Indeed, based on the timing of sensory neuropathology and other studies using our model (Wodarski et al., 2018; Bagdas et al., 2020), 6 weeks of Tat induction in our tg mice is thought to approximate the initial stages of infection in PWH. Thus, we anticipate that similar reductions in PPI will be apparent in our Tat-tg model with more prolonged exposure. Other preclinical models (HIV-tg rats and gp120-tg mice) also do not display consistent results, e.g., some studies show a HIV-protein-induced reduction in PPI, whereas others show no change (Moran et al., 2013; Henry et al., 2014; Roscoe et al., 2014; Bachis et al., 2016; McLaurin et al., 2017a,b, 2018; Roberts et al., 2021). It is anticipated that our results would differ from these because of the involvement of different HIV proteins (or different combinations of proteins), and we predict each viral protein will affect PPI differently or not at all.

Alterations in the stress response induced by the twice daily injections before testing (Drude et al., 2011) done in the present study [vs. single daily (Paris et al., 2015) or no (Nass et al., 2020) injections] may indicate a role of the hypothalamic-pituitary-adrenal (HPA) axis. Tg mice that chronically overexpress corticotrophin releasing factor (CRF) within the CNS display reduced acoustic startle response (Dirks et al., 2002). Alterations in CRF (de Guglielmo et al., 2019) and glucocorticoids (Koob, 2020) can contribute to the negative emotional states, hyperkatifeia, and heightened HPA axis stress, which are often associated with opioid withdrawal. While Tat can disrupt this system (Paris et al., 2020; Salahuddin et al., 2020a,b), its actions in the amygdala are less well studied. However, we previously found that CRF levels within the amygdala of Tat(+) mice correlate inversely with sociability in mice (Nass et al., 2021), suggesting a role for amygdalar CRF in modulating Tat-induced behavioral deficits.

HIV-1 Tat exposure for 8 weeks also decreased dendritic spine density on glutamatergic pyramidal neurons in the BLA (input nuclei), but not CeA GABAergic neurons (output nuclei) of the amygdala (summarized in Figure 7). These data align with a previous report indicating a decrease in the expression of genes related to postsynaptic density formation in the amygdala of HIV-tg rats (Nesil et al., 2015). However, these results are surprising given that stress and anxiety tend to increase BLA dendritic spine density and arborization (McEwen et al., 2016; Zhang et al., 2018) and anxiety-like behavior in gp120-tg mice is associated with increased dendritic spine density within the BLA (Bachis et al., 2016). Although Tat exposure also induces anxiety-like behavior (Paris et al., 2014; Hahn et al., 2016; Salahuddin et al., 2020a), these disparate results may be due to the etiology of the stressor (external stressors vs. neurodegenerative pathology) or the combination of an external (injection/handling) and internal (Tat) stressor in the present study (Steimer, 2002; Levenson et al., 2014). Indeed, BLA pyramidal neuron dendritic complexity is decreased in APP/PS1-tg mice, a model of AD (Knafo et al., 2009; Guo et al., 2017). Further, although Tat exposure can induce HPA axis stress (Paris et al., 2020; Salahuddin et al., 2020a,b), these effects were potentiated by a 15-min external swim stressor (Salahuddin et al., 2020a), suggesting both pathology and external stressors can influence glucocorticoid and CRF levels in Tat-tg mice. Synaptodendritic injury is thought to underly the development of HAND (Masliah et al., 1997; Ellis et al., 2007), and Tat disrupts excitatory and inhibitory synaptic connections in multiple brain regions that project to the BLA, including the hippocampus (Fitting et al., 2013; Marks et al., 2016, 2021) and medial prefrontal cortex (Xu et al., 2017; Nass et al., 2020). These pathological inputs could also disrupt synaptic connections within the amygdala microcircuitry. However, Tat expression in an alternative model (Kim et al., 2003) is also known to decrease amygdalar volume (Carey et al., 2013) and trigger microgliosis indicative of persistent neuroinflammation in the amygdala (Paris et al., 2015), suggesting a direct role of Tat in amygdalar pathology.

Neuroadaptations within the extended amygdala mediate the negative emotion/aversion and stress learning associated with opioid withdrawal (Koob and Volkow, 2016; Koob, 2021). Similar to previous studies (Harris and Gewirtz, 2004; Harris et al., 2004; Cabral et al., 2009; Lee et al., 2017), testing 4 h after the final dose of 2 weeks of repeated escalating morphine increased the acoustic startle response in mice regardless of Tat exposure, suggesting an anxiogenic negative affect response that is characteristic of opioid dependence (Harris and Gewirtz, 2005). The BLA and CeA are highly enriched in MOR-expressing neurons (Zhu and Pan, 2005; Merg et al., 2006; Wilson and Junor, 2008; Zhang et al., 2015). While postsynaptic MOR activation by endogenous Met-enkephalin in subsets of GABAergic intercalated cells within the amygdala results in a net anxiogenic output from the CeA (Blaesse et al., 2015; Winters et al., 2017), stimulation of presynaptic δ opioid receptors (or MOR) at other locations within the microcircuitry may be anxiolytic (Winters et al., 2017). Therefore, the lack of morphine-induced changes in amygdalar spine density in the present study, despite an anxiogenic startle response may be due to their predominant expression on GABAergic neuronal subtypes (Figure 6). However, the glutamatergic AMPA receptor antagonist NBQX infused directly into the amygdala, attenuates the acute spontaneous opioid withdrawal-induced potentiation of the startle response (Harris et al., 2006), suggesting a role for inhibitory and excitatory imbalances within the amygdala in opioid dependence-induced stress/anxiety-like behavior.

We previously found that Tat markedly reduces MOR-mediated G-protein activation in the amygdala while at the same time elevating β-arrestin-2 levels and increasing β-arrestin-2-MOR co-immunoprecipitation (Hahn et al., 2016). This suggests that Tat enhances MOR desensitization. Although few morphine and Tat interactions were observed in this study, combined morphine and Tat exposure decreased habituation and PPI to the startle response, perhaps related to the increased anxiety-like behavior that we previously observed with decreased Tat-dependent MOR-mediated G-protein activation in the amygdala (Hahn et al., 2016). Although the findings of Winters et al. (2017) might predict Tat-induced MOR desensitization to be anxiolytic, Tat likely affects each site within the microcircuitry of the amygdala differently, as shown by differing effects on spine density of pyramidal neurons in the BLA vs. medium spiny-type neurons in the CeA (Figure 3). Tat-induced synaptic dysfunction is regional and dependent on neuron subtype across the CNS (Fitting et al., 2010, 2013; Marks et al., 2016, 2021; Schier et al., 2017; Cirino et al., 2020; Nass et al., 2020). In addition, the behavioral output of MOR activation appears to be anxiolytic or anxiogenic depending on the threat context (e.g., discrete vs. potential threats) (Zhu and Pan, 2005), suggesting that the somewhat unexpected anxiety-like behavior might be due to the behavioral assay used.

The amygdala is highly susceptible to the accumulation of pathological misfolded proteins including Aβ and tau that are the hallmark of AD and other neurodegenerative disorders (Nelson et al., 2018). The complexation of oligomers from monomers promotes the formation of neurotoxic aggregates of the Aβ peptide. Amyloid precursor protein/presenilin 1 (APP/PS1) tg mice accumulate Aβ within the CNS, which is associated with decreased dendritic length, branching, and spine density in the lateral amygdala (Knafo et al., 2009). Further, CRND8-tg mice, which have a mutation in APP leading to CNS Aβ accumulation exhibit increased acoustic startle response and decreased PPI (McCool et al., 2003). Unlike the dorsal striatum, in which medium spiny neuronal dendrites undergo frank degeneration (varicosity formation and fragmentation) after 1–2 weeks of Tat and morphine exposure (Fitting et al., 2010; Schier et al., 2017), the amygdalar response was limited to reductions in spine densities only within the BLA, which coincided with elevated levels of Aβ monomers (summarized in Figure 7). It is likely that Tat-induced neuronal damage in the amygdala will worsen with more prolonged exposure as Aβ monomers are complexed into oligomers.

In contrast to Tat, morphine exposure alone increased both monomeric and oligomeric Aβ peptides within the amygdala. This supports recent research suggesting that morphine increases Aβ in human astrocytes *in vitro* (Sil et al., 2021). However, other studies show that morphine might be protective against Aβ toxicity in neurons *in vitro* by increasing nitric oxide (Pak et al., 2005) and estradiol production (Cui et al., 2011). Given that amyloid pathology is seen in the brains of chronic opioid users (Ramage et al., 2005; Anthony et al., 2010; Kovacs et al., 2015), the differences between the detrimental vs. protective effects of morphine may be due to MOR-induced inflammation in the presence of neurotoxic proteins *via* glial activation (Zou et al., 2011; Kim et al., 2018) that was not present in the *in vitro* neuron cultures (Pak et al., 2005; Cui et al., 2011). Morphine exposure also increased amygdalar levels of the pathological phospho-tau isotype Ser396 and tended to increase Thr205. Similarly, morphine increased Ser396 p-tau levels in rat cortical neurons *in vitro* (Cao et al., 2013) and mouse PFC *in vivo* (Ohene-Nyako et al., 2021). Tat co-exposure tended to only modestly augment the effects of morphine in the amygdala, which differed from findings in the striatum where Tat and morphine have been found to interact at the Ser396 residue of p-tau (Ohene-Nyako et al., 2021). However, the increase in Aβ monomers and decrease in habituation and PPI to the startle response was driven by Tat(+) mice administered morphine—suggesting some interaction that may be dependent on exposure time or specific to particular amygdalar nuclei. Regardless, the results suggest that the neuropathological effects of Tat and morphine are largely independent of one another in the amygdala, though the possibility that more subtle interactions may occur at highly specific sites within amygdalar microcircuitry cannot be excluded. Lastly, we find repeated (2–6 week) exposure to morphine by itself may be sufficient to cause neurodegeneration in some brain regions (Ohene-Nyako et al., 2021). This is perhaps not surprising since emerging evidence indicates OUD can promote cognitive decline (Dublin et al., 2015; Sanjari Moghaddam et al., 2021) and an AD-like pathology (Ramage et al., 2005; Anthony et al., 2010; Kovacs et al., 2015) despite few studies examining the CNS histopathologically following prolonged opioid exposure.

## Conclusion

Our results provide the first empirical evidence that repeated opioid exposure (2 weeks of morphine) increases amygdalar levels of pathological Aβ and phospho-tau isotype Ser396, suggesting opioids can induce neurodegeneration in the amygdala, a key brain region in addictive behavior. The pathologic accumulation of tau and Aβ were accompanied by an increase in the acoustic startle response. In contrast, Tat by itself decreases BLA pyramidal neuron dendritic spine density and the acoustic startle response. Unlike other CNS regions, such as the striatum, interactions between Tat and morphine were relatively limited. Co-exposure decreased habituation and PPI to the startle response and potentiated the amygdalar accumulation of monomeric Aβ. Overall, Tat and morphine appear to differentially induce amygdalar injury and alter associated startle response, indicating an underexplored role of the amygdala in HAND and OUD neuropathology.

## Data Availability Statement

The raw data supporting the conclusions of this article will be made available by the authors, without undue reservation.

## Ethics Statement

The animal study was reviewed and approved by the Virginia Commonwealth University Animal Care and Use Committee.

## Author Contributions

SN, PK, and KH conceptualized, designed the experiments, and drafted the article for important intellectual content. SN, MO-N, and YH acquired the data. SN, MO-N, and KH carried out data analysis and interpretation. All authors participated in proofing, revising, and approving the final version of the manuscript.

## Conflict of Interest

The authors declare that the research was conducted in the absence of any commercial or financial relationships that could be construed as a potential conflict of interest.

## Publisher’s Note

All claims expressed in this article are solely those of the authors and do not necessarily represent those of their affiliated organizations, or those of the publisher, the editors and the reviewers. Any product that may be evaluated in this article, or claim that may be made by its manufacturer, is not guaranteed or endorsed by the publisher.
